# Supplementary material for: 5-Arylidenerhodanines as P-gp Modulators: An Interesting Effect of the Carboxyl Group on ABCB1 Function in Multidrug-Resistant Cancer Cells
Source: Int J Mol Sci. 2022 Sep 16;23(18):10812. doi: 10.3390/ijms231810812 (PMC9503420; doi:10.3390/ijms231810812)
Supplement: Supplementary file 1 [file ijms-23-10812-s001.zip › ijms-1867989-supplementary.pdf]

Supplementary Materials

Table S1. The interplanar angles (°) between the planes containing rings.

Planes	3	7	11
rhodanine/benzylidene	mol. A 6.0(1)<br>mol. B 6.3(1)	mol. A 18.1(1)<br>mol. B 15.7(1)	7.3(1)
benzylidene/phenyl 1	mol. A 19.4(1)<br>mol. B 50(1)	mol. A 24.5(1)<br>mol. B 20.6(1)	70.24(5)
benzylidene/phenyl 2	-	-	56.06(6)
phenyl 1/ phenyl 2	-	-	85.18(5)

Table S2. Parameters of intermolecular interaction in the crystals of **3**, **7** and **11**.

Cmpd.	D-H···A	H···A (Å)	D···A (Å)	D-H-A (°)	Symmetry code
**3**	O3A-H3A···O2B	1.78(3)	2.674(2)	173(3)	-x+1, -y+1, -z+1
	O3B-H3B···O2A	1.78(4)	2.627(2)	170(4)	-x+1, -y+1, -z+1
	C6A-H6A···O1A	2.45	3.339(2)	159.7	-x, -y+2, -z+1
	C6B-H6B···O1B	2.32	3.193(2)	156.6	-x+1, -y+2, -z+1
	C15B-H15B···S1B	2.99	3.572(2)	122.3	-x+2, -y+3, -z+2
	C17B-H17B···S2A	2.96	3.667(2)	134.3	x, y+1, z
	C18B-H18B···O3B	2.53	3.400(3)	155.7	x, y+1, z
	C19B-H19D···O1A	2.66	3.333(2)	126.8	x+1, y, z
**7**	O3A-H3A···O2B	1.80(4)	2.633(2)	173(4)	-x, -y-1, -z+1
	O3B-H3B···O2A	1.75(5)	2.667(2)	169(4)	-x, -y-1, -z+1
	C6A-H6A···O1B	2.60	3.374(3)	140.8	-x+1, -y, -z+1
	C13A-H13A···O3A	2.58	3.391(3)	141.0	x+1, y+1, z
	C13A-H13C···O1A	2.63	3.460(3)	143.7	-x+2, -y, -z+1
	C13B-H13D···O3B	2.56	3.314(3)	135.1	x+1, y+1, z
	C18A-H18A···O3A	2.92	3.625(3)	134.2	-x+2, -y+1, -
	C21A-H21A···O3A	2.57	3.453(3)	151.0	-x+1, -y-1, -z+1
**11**	O3-H3···O2	1.76(3)	2.645(2)	177(3)	-x, -y+1, -z+2
	C9-H9···O1	2.62	3.392(2)	139.0	-x+2, -y+1, -z+1
	C14-H14···S2	2.98	3.673(2)	131.4	-x+2, -y, -z+1
	C16-H16···O2	2.70	3.488(2)	140.2	-x+2, -y+1, -z+1
	C17-H17···O3	2.78	3.541(2)	137.2	-x+2, -y+1, -z+1
	C18-H18···O2	2.49	3.353(2)	151.8	-x+1, -y+1, -z+1
	C21-H21···S2	3.02	3.583(2)	106.2	-x+1, -y, -z+1
	C24-H24···O3	2.61	3.466(2)	149.8	x+1, y, z-1

Table S3. Docking scores, MM/GBSA free energy of binding and interactions observed for complexes of the *h*Pgp homology model with the docked ligands.

Compound	XP GScore	IFD Score	MMGBSA ΔG kcal/mol	H-bond	π···π	Interacting residues within 4Å
**Verapamil**	-9.387	-2651.60	-87.19	Tyr307<br>Tyr953	Phe983	TM1: Leu65, Met69, Phe72<br>TM5: Ile306, Tyr307, Tyr310<br>TM6: Phe335, Phe336, Phe343<br>TM7: Gln725, Phe728, Ala729, Phe732<br>TM8: Phe759<br>TM9: Asn842<br>TM11: Tyr953, Phe957<br>TM12: Leu975, Phe978, Ser979, Phe983, Met986, Ala987
**11**<br>pose **Ia**	-11.031	-2660.88	-87.96	Gln946<br>Tyr950	Phe336<br>Phe732<br>Phe983	TM1: His61, Leu65, Met68, Met69<br>TM5: Tyr310<br>TM6: Leu332, Phe336<br>TM7: Phe728, Ala729, Phe732, Ile736<br>TM11: Gln946, Met949, Tyr950, Tyr953<br>TM12: Leu975, Phe978, Ser979, Val982, Phe983, Met986
**11**<br>pose **Ib**	-9.028	-2659.08	-68.07	Gln347	Phe72<br>Phe732<br>Phe978<br>Phe983	TM1: Leu65, Met69, Phe72<br>TM3: Thr199<br>TM6: Leu332, Phe336, Ile340, Phe343, Ser344, Gln347<br>TM7: Phe728, Ala729, Phe732, Ile736<br>TM11: Tyr953<br>TM12: Leu975, Phe978, Ser979, Val982, Phe983, Met986

Table S4. Docking scores, MM/GBSA free energy of binding and interactions observed for complexes of the cryo-EM structure 7O9W with the docked ligands.

Compound	XP GScore	IFD Score	MMGBSA ΔG kcal/mol	H-bond	π···π	Interacting residues within 4Å
**Verapamil**	-10.974	-2243.60	-102.10	Tyr307<br>Tyr953<br>Gln990 x 2	Phe983	TM1: Met69, Phe72<br>TM5: Phe303, Ile306, Tyr307, Tyr310<br>TM6: Phe336<br>TM7: Leu724, Gln725, Phe728, Ala729, Phe732<br>TM8: Phe770<br>TM9: Asn842<br>TM11: Tyr953, Phe957<br>TM12: Leu975, Phe978, Ser979, Phe983, Met986, Ala987, Gln990, Val991
**11**<br>pose **II**	-9.328	-2239.03	-92.06	Gln347	Phe336<br>Phe978	TM1: Leu65, Met69, Phe72, Gly73, Thr76<br>TM5: Tyr310<br>TM6: Leu332, Thr333, Phe336, Leu339, Ile340, Phe343, Ser344, Gln347<br>TM7: Phe728, Phe732<br>TM11: Tyr953, Phe957<br>TM12: Leu975, Phe978, Ser979, Phe983

Table S5. Comparison of predicted selected physicochemical properties.

No.	Log P	Log S
**1**	3.69	-5.47
**2**	3.90	-5.27
**3**	4.04	-5.27
**4**	4.67	-5.37
**5**	3.78	-5.47
**6**	3.66	-5.27
**7**	3.80	-5.28
**8**	4.15	-5.41
**9**	5.19	-5.80
**10**	5.17	-5.60
**11**	5.17	-5.57
**12**	5.33	-5.64

Values predicted using ALOGPS 2.1 applet of the Virtual Computational Chemistry Laboratory (http://www.vclab.org).
